# VALOR2: characterization of large-scale structural variants using linked-reads

**DOI:** 10.1186/s13059-020-01975-8

**Published:** 2020-03-19

**Authors:** Fatih Karaoğlanoğlu, Camir Ricketts, Ezgi Ebren, Marzieh Eslami Rasekh, Iman Hajirasouliha, Can Alkan

**Affiliations:** 1 Department of Computer Engineering, Bilkent University, Ankara, 06800 Turkey; 2 Tri-Institutional Computational Biology & Medicine Program, Cornell University, 1300 York Ave, New York, 10065 NY USA; 3 Graduate Program in Bioinformatics, Boston University, 24 Cummington Mall, Boston, 02215 MA USA; 4 Department of Physiology and Biophysics, Institute for Computational Biomedicine, Weill Cornell Medicine, 1300 York Ave, New York, 10065 NY USA; 5 Englander Institute for Precision Medicine, The Meyer Cancer Center, Weill Cornell Medicine, 1300 York Ave, New York, 10065 NY USA; 6 Bilkent-Hacettepe Health Sciences and Technologies Program, Bilkent University, Ankara, 06800 Turkey

**Keywords:** Structural variation, Linked-reads, WGS

## Abstract

Most existing methods for structural variant detection focus on discovery and genotyping of deletions, insertions, and mobile elements. Detection of balanced structural variants with no gain or loss of genomic segments, for example, inversions and translocations, is a particularly challenging task. Furthermore, there are very few algorithms to predict the insertion locus of large interspersed segmental duplications and characterize translocations. Here, we propose novel algorithms to characterize large interspersed segmental duplications, inversions, deletions, and translocations using linked-read sequencing data. We redesign our earlier algorithm, VALOR, and implement our new algorithms in a new software package, called VALOR2.

## Background

Alterations of DNA content and organization larger than 50 bp, commonly referred to as genomic structural variations (SVs) [1], are among the major drivers of evolution [2, 3] and diseases of genomic origin [4]. Despite decades of research, they remain difficult to accurately characterize contributing to our lack of full understanding of the etiology of complex diseases, termed *missing heritability* [5].

High-throughput sequencing (HTS) technologies are widely employed to discover and genotype various classes of SVs since their inception [6–13]. However, effectiveness has been limited by either very short read lengths (e.g., Illumina) or high error rates (e.g., PacBio and Oxford Nanopore). The human genome complexity further contributes to our lack of full characterization of structural variants, especially large-scale duplications and balanced rearrangements (inversions and balanced translocations) due to the repetitive and duplicated sequence at the SV breakpoints [14]. Despite high error rates and high requirement for DNA input, long reads offer improvement in complex SV discovery, either used alone [15, 16] or when integrated with standard short-read sequencing data [17].

Recently the linked-read sequencing method such as the 10x Genomics system (10xG), transposase enzyme linked long-read sequencing (TELL-Seq), and single-tube long fragment read (stLFR) was introduced as an alternative method to generate highly accurate Illumina short-read data with additional long-range information [18]. In linked-read sequencing, large DNA molecules (typically 10–100 kbp) are barcoded and randomly separated into a very large number of partitions (here, we term these partitions “pools”). For example, in the 10xG system, each pool contains roughly 2–30 large molecules, and the number of pools is typically over a million. These pools are then sequenced at very low coverage (∼0.1×) using the standard Illumina platform. Shared barcodes among Illumina read pairs show them as generated from the same pool. Since each pool is diluted to contain only a very small fraction of the input DNA, the probability of barcode collision is negligible [19]. For example, assuming 20 molecules per pool and an average size of 30 kbp per molecule, each pool on average contains only $\frac {1}{5000}$ of the haploid human genome. Linked-reads then can be used to “reconstruct” large molecules that originate from the same haplotype. Furthermore, linked-read sequencing makes it possible to obtain very high physical coverage with the cost of generating moderate sequence coverage data^1^.

The ability of extracting long-range information from accurate and inexpensive but short-read sequencing data makes linked-read sequencing attractive for various applications [13]. It has been used for genome scaffolding [20], haplotype-aware assembly [18, 21, 22], metagenomics [23], single-cell transcriptome profiling [24, 25] and regulatory network clustering [26], haplotype phasing [18, 21, 27], and genome structural variation discovery [19, 28–30].

Linked-read techniques for genomic structural variation discovery include VALOR [28], Long Ranger [29], and GROC-SVs [30]. VALOR was the first algorithm that used “split molecule” signature, similar to the commonly used split read signature [31], together with traditional read pair signature [1, 8, 32] to characterize large (> 500 kbp) inversions. Split molecules are defined as large molecules that span an SV breakpoint, and therefore mapped as two disjoint intervals to the reference genome.

Long Ranger [29] is a comprehensive software package developed by 10x Genomics, for the purpose of barcode-aware read alignment (Lariat module) and resolving full-scale human germline genome variation, while GROC-SVs is an optimized tool for somatic and complex SVs in cancer genomes. Both Long Ranger and GROC-SVs employ a novel idea to utilize discordance in expected “barcode coverage” as well as barcode similarities across distant locations for potential large-scale SV signals. In addition, GROC-SVs [30] performs local assembly on barcoded reads to detect large complex events that are between 10 and 100 kbp with breakpoint resolution.

Despite the aforementioned advances in SV discovery using various technologies, detecting complex SV such as balanced rearrangements (i.e., inversions and translocations), and segmental duplications (SDs) remains challenging due to mapping ambiguity. Note that it is still possible to identify increase in SD copy number using read depth signature [33, 34]; however, no linked-read-based method yet exists to *anchor* a new SD (i.e., find their insertion locations). We note that the TARDIS algorithm [35] can locate new SDs; however, it is developed for short-read sequencing data only; therefore, it can find only short duplications (up to 10 kbp) copied to a distance of up to 50 kbp.

Here, we present *novel algorithms* to discover deletions, inversions, translocations, and large (> 40 kbp) direct and inverted interspersed SDs using linked-read sequencing data. We redesign and extend upon VALOR and use split molecule and read pair signatures to detect SDs and estimate the insertion sites of the new SD paralogs, and further include read depth signature to filter potential false positives caused by incorrect mappings. We implemented our new algorithms as the VALOR2 software package. Briefly, VALOR2 differs from the former version of VALOR through (1) it can characterize segmental duplications in both direct and inverted orientation, (2) it can discover translocations and deletions, (3) it incorporates read depth information to improve predictions and reduce false calls, (4) it provides full support to alignment files (i.e., BAM) generated from 10xG linked-read data sets, and (5) provides a 10-fold speed up in run time (data not shown).

Using simulated data sets, we show that VALOR2 achieves high precision and recall (85% and 83%, respectively) for segmental duplications, 83% and 60% for large inversions, 91% and 87% for deletions, and 100% and 71% for translocations. We also applied VALOR2 to the genomes of NA12878 and a Yoruban trio (NA19238, NA19239, NA19240) in addition to two haploid genomes (CHM1 [18], CHM13 [36]) sequenced with the 10xG platform.

## Methods

**Fig. 1:**
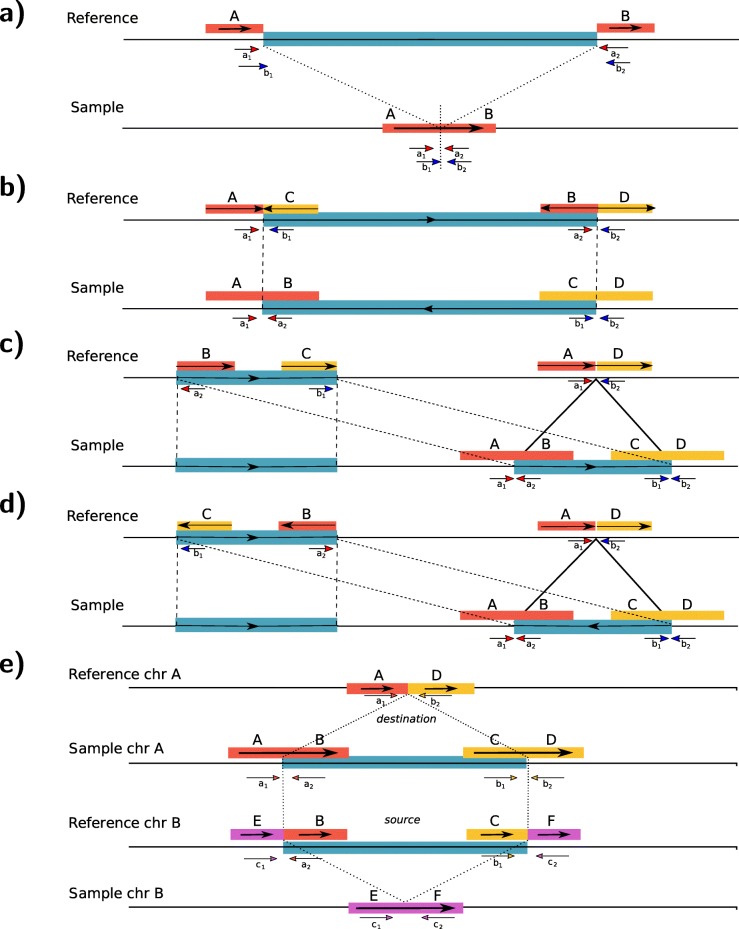
Split molecule and read pair sequence signatures used in VALOR2. **a** Deletion. **b** Inversion. **c** Interspersed duplication in direct orientation. **d** Inverted duplication. **e** Translocation. Note that **e**, shows only non-reciprocal translocations. For reciprocal translocations please refer to Additional file 1: Figure S1). In each case, the large molecules that span the SV breakpoints are split into two mapped regions. Note that it is not possible to determine the mapped strand of the split molecules shown here. In **e**, the section including B and C is moved to between A and D. We do not show the inverted translocations here for simplicity. From the perspective of the reference genome (i.e., mapping), A, B, C, D, E, and F are defined as *submolecules*; A/B, C/D, and E/F pairs are *candidate splits*; and A/B-C/D quadruple is a *split molecule pair*

We have previously described an earlier version of VALOR2 that uses split molecules and read pair signature to detect inversions [28]. Here, we describe novel formulations, algorithms, and optimizations to characterize large (> 80 kbp) inversions, deletions (> 100 kbp), translocations (> 100 kbp), and *segmental duplications* (> 40 kbp) in both direct and inverted orientations. We depict the split molecule and read pair sequence signatures for these types of large SVs in Fig. 1.

### Glossary

Here, we define several terms that we use in this manuscript:

- *Molecule*: a large molecule (30–50 kbp) that was barcoded and pooled using a linked-read platform. Here, we refer to as the physical entity.
- *Submolecule*: a molecule identified in silico by the VALOR2 algorithm by analyzing the read map locations.
- *Candidate split*: a pair of submolecules with the same barcode that potentially signal single breakpoint of an SV event.
- *Split molecule pair*: a pair of candidate splits with different barcodes that potentially signal the different breakpoints of the same SV event.

### Overview of the VALOR2 algorithm

VALOR2 depends on only the alignment files (i.e., BAM) with the necessary barcode information generated with Long Ranger/Lariat, BWA-MEM, or a similar read mapper. Briefly, VALOR2 first tries to identify the underlying large molecules separately for each barcode, which we call *submolecules*. In this step, we do not consider reads that map to satellite regions, and we discard very short submolecules. Two identified submolecules are paired together (called *candidate splits*) if the summation of their span is ≤*μ*_molecule_+3*σ*_molecule_ where *μ*_molecule_ is the average and *σ*_molecule_ is the standard deviation of the inferred submolecule sizes. Next, VALOR2 removes those candidate splits with no read pair support. VALOR2 then (1) matches candidate splits with different barcodes that are likely to signal individual breakpoints of the same SV event; (2) filters out candidates with low read pair support, additionally it discards those that signal a deletion or duplication event without read depth support; and (3) models the split molecule pairs as vertices in a graph and approximately discovers the maximal quasi cliques for each connected component of this graph. In this graph, edges represent overlap (i.e., “agreement”) between two split molecule pairs. Finally, VALOR2 reports SVs that are supported by more than a threshold of split molecules.

Below, we present the details for each step in the VALOR2 algorithm.

### Molecule recovery

The first step of the VALOR2 algorithm involves identification (or, recovery) of the large molecules from mapped data. Initially, we call the intervals returned by this recovery as *submolecules*. For this purpose, we use a sliding window approach to greedily group reads with the same barcode which are mapped in close proximity (Additional file 1: Algorithm S1). Here, we only consider concordantly mapped read pairs, and we take the full span of a read pair as a *fragment*. For each barcode, we scan each chromosome and merge together fragments if they are within a user-defined distance *T*, or if a new fragment is within distance *Q* from the leftmost fragment in a re-identified submolecule. We use *Q*=2·*μ*_molecule_ and *T*=*μ*_molecule_/4 by default^2^, determined by parameter sweeping. Finally, we remove very short submolecules (< 3 kbp by default) that correspond to less than 10% of expected average molecule size from consideration.

### Candidate split matching

We first record all pairs of submolecules that share the same barcode and map to the same chromosome as *candidate splits* and then compare all possible pairs of candidate splits across different barcodes (termed *split molecule pairs*) to find those that signal a structural variation (see Fig. 1 for the depiction of candidate splits and split molecule pairs). We limit inversion predictions and the duplication size by the largest inversion size we can find in the literature [37] (≈ 7 Mbp). Next, we test whether the split molecule pairs are supported by read pair signature (Fig. 1). Here, we require at least 3 read pairs to signal the same SV event, and we remove candidate splits with insufficient support from consideration.

#### Candidate splits for translocations

While it is possible to exhaustively test all pairs of candidate splits for intra-chromosomal events, it is infeasible to follow the same approach for inter-chromosomal variants. This is due to the relatively high number of distinct molecules sharing the same barcode (up to 30) and very high number of barcodes (up to 4 million). To overcome this issue, we first use discordant read pairs as anchors and attach two other submolecules with the same barcode that map close to each end (Additional file 1: Figure S2).

### Clustering using SV graph

**Fig. 2:**
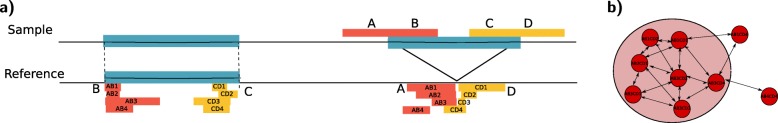
Building the SV graph from split molecule pairs for an interspersed duplication. **a** Four pairs of split molecules that signal the event. **b** Corresponding SV graph, where each vertex denotes a pair of submolecules that signal the SV, and edges show “agreement” between pairs. The shaded area corresponds to the quasi-clique selected as representative of the putative SV

We construct an SV graph *G* as follows (Fig. 2). We denote each remaining split molecule pair as a vertex in *G*, and we create an edge between two vertices if their corresponding split molecule pairs signal the same SV event. Finally, on the resulting graph, we find clusters of read pair-supported split molecule pairs by approximately solving the maximal clique problem using the quasi-clique formulation [38]. Here, a quasi clique is defined as an approximate clique with *V* vertices and *γ*·(|*V*|2) edges, where *γ* is a user-defined parameter, which we set to *γ*=0.6 by default. Each quasi clique defines a putative SV event.

We identify inversion and deletion breakpoints with two coordinates, duplications, and translocations with three coordinates. Third breakpoint denotes the insertion coordinates given within a confidence interval.

### Molecule depth filtering

**Fig. 3:**
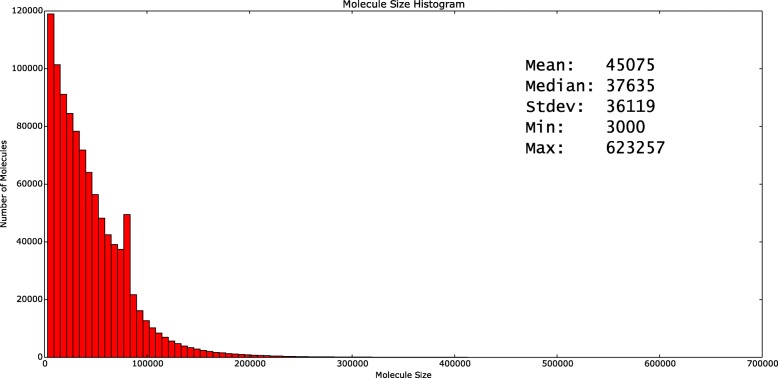
Molecule size histogram mapped to chromosome 1 as observed in the linked-read sequencing data generated from the genome of the NA12878 sample [18]

Although there are only a small number of molecules that share the same barcode (2–30), it is still possible that two or more different molecules originate from the same chromosome. Additionally, the molecule sizes do not follow Gaussian, Poisson, or a similar distribution (Fig. 3); thus, it is not possible to distinguish true split molecules from “normal” but short molecules. The read pair sequence signature is not entirely reliable either due to the mismapping artifacts within or around repeats and duplications. We, therefore, apply additional filtering on duplication calls based on “molecule depth.” We reason that the number of molecules that originate from segmental duplications must be higher than the genome-wide average, similar to the traditional read depth signature [33, 39]. In this step, we first calculate the average molecule depth (*μ*_depth_) and standard deviation (*σ*_depth_) in the entire genome. We then discard segmental duplication predictions with molecule depth <*μ*_depth_+*σ*_depth_, deletion predictions with molecule depth >0.5*μ*_depth_+0.5*σ*_depth_, and translocation predictions with molecule depth outside *μ*_depth_±1.5*σ*_depth_ at the source.

## Results

We tested VALOR2 using both simulated and real data sets to compare the precision and recall rates of VALOR2 with the state-of-the-art tool that use linked-read sequencing (Long Ranger [29]), three tools that use only short-read WGS data sets (DELLY [40] LUMPY [11], TARDIS [12, 35]), and one that uses long read WGS data sets (Sniffles [41]). For LUMPY, we used the smoove wrapper as recommended by the authors. We also tried to run GROC-SVs; however, the tool crashed due to excessive memory usage.

Among these tools, VALOR2 and TARDIS are the only tools that can characterize interspersed duplications. However, the size range of variants that they can detect is complementary. VALOR2 aims to find duplications larger than 40 kb copied to > 80 kb away from the source, where TARDIS can only detect duplications that are copied within 50 kb from the source; therefore, we removed TARDIS from comparisons of segmental duplication predictions. Since there is no comparable tool to our knowledge, we only provide VALOR2 results on interspersed duplications. We compared inversion and deletion prediction performance of VALOR2 with LUMPY, DELLY, TARDIS, Sniffles, and Long Ranger. Similarly, we compared the translocation predictions with LUMPY, DELLY, and Long Ranger since TARDIS and Sniffles do not currently support translocation discovery. As we designed VALOR2 as a complementary method, we also provide results of union and intersection of VALOR2 and Long Ranger SV calls.

### Simulation experiments

We used VarSim [42] to generate a simulated diploid human genome. We note that VarSim randomly selects SNVs, indels, and SVs from a database of *known* variants and inserts them into the simulated genome. Our simulation included variants of various lengths and types: 2.8 million SNPs, ≈ 195,000 indels, and ≈ 5000 SVs (> 50 bp, up to 6 Mbps). We found that VarSim only generates tandem duplications and does not simulate translocations; therefore, we randomly changed a subset of simulated tandem duplications to interspersed duplications and non-reciprocal translocations (by deleting the source copy) in the simulated VCF file, assigned random insertion breakpoints, and then applied changes to the reference. We then generated Illumina WGS reads using ART [43] and PacBio long reads using PBSim [44] at 40 × depth of coverage and 10xG linked-reads at 50 × coverage using LRSim [45]. The 10xG linked-read simulation has extra coverage to account for the barcode sequences that are part of the read and other losses as also described in [29].

Auxiliary files released with the current version of VarSim only support the human reference genome build 37 (GRCh37); therefore, we mapped the simulated reads to GRCh37 using BWA-MEM [46] for Illumina, NGMLR [41] for PacBio (as recommended by Sniffles authors), and Long Ranger for 10xG data sets. We then applied the standard BAM processing that includes sorting with SAMtools [47] and marking duplicates with Sambamba [48]. We used VALOR2 and Long Ranger to generate SV call sets from the 10xG simulation, and DELLY, LUMPY, and TARDIS to call variants using the Illumina simulation, and Sniffles using the PacBio simulation (see Additional file 1: Table S1 for version numbers for tools and respective command lines). We limited our comparison to only large SVs (> 80 kbp for inversions, > 40 kbp for duplications (> 100 kbp for deletions and translocations), and we required > 50% reciprocal overlap between the simulation and the prediction for SVs using BEDtools [49]. We also require the inferred insertion breakpoint is within a distance of *μ*_molecule_/2 (in simulation experiments *μ*_molecule_ = 50 kbp) of the simulated breakpoint to consider a duplication to be correctly predicted.

We present the prediction performance of the tools we tested in Table 1. We found that VALOR2 is able to correctly predict > 82% of large duplications (inverted and direct combined) and 60% of large inversions, while maintaining 84–86% precision for duplications and 83% precision for inversions. Long Ranger, the other algorithm that used linked-reads, demonstrated the same recall rate (60%) of the inversions with lower precision (73%).

**Table 1:** Prediction performance evaluation using simulated structural variants

**Variant**	**Tool**	**# Sim.**	**# Pred.**	**TP**	**FP**	**FN**	**Pr.**	**Rec.**	**F1**
Duplications (direct)	VALOR2	111	103	89	14	22	**0.86**	**0.80**	**0.83**
Duplications (inverted)	VALOR2	49	51	43	8	6	**0.84**	**0.88**	**0.86**
Inversions	VALOR2	90	65	54	11	36	0.83	0.60	0.70
	VALOR_1_	90	63	47	13	43	0.78	0.52	0.63
	LUMPY/smoove	90	35	27	7	63	0.79	0.30	0.44
	DELLY	90	358	39	293	51	0.12	0.43	0.18
	TARDIS	90	43	34	1	56	0.97	0.38	0.54
	Sniffles	90	787	72	603	18	0.11	**0.80**	0.19
	Long Ranger	90	75	54	20	36	0.73	0.60	0.66
	Long Ranger ∪ VALOR2 ^*‡*^	90	102	70	31	20	0.69	0.78	**0.73**
	Long Ranger ∩ VALOR2	90	38	38	0	52	**1.00**	0.42	0.59
Deletions	VALOR2	85	81	74	7	11	0.91	0.87	0.89
	LUMPY/smoove	85	292	66	226	19	0.23	0.78	0.35
	DELLY	85	496	72	424	13	0.15	0.85	0.25
	TARDIS	85	152	70	82	15	0.46	0.82	0.59
	Sniffles	85	467	72	395	13	0.15	0.85	0.26
	Long Ranger	85	262	79	175	6	0.31	0.93	0.47
	Long Ranger ∪ VALOR2 ^*‡*^	85	270	163	185	3	0.47	**0.98**	0.63
	Long Ranger ∩ VALOR2	85	84	79	5	6	**0.94**	0.93	**0.93**
Translocations	VALOR2	38	27	27	0	11	**1.00**	0.71	0.83
	LUMPY/smoove	38	4	2	2	36	0.50	0.05	0.10
	DELLY	38	116	30	86	8	0.26	0.79	0.39
	Long Ranger	38	29	26	3	12	0.90	0.68	0.78
	Long Ranger ∪ VALOR2 ^*‡*^	38	38	53	3	3	0.95	**0.95**	**0.95**
	Long Ranger ∩ VALOR2	38	18	18	0	20	**1.00**	0.47	0.64

We evaluate the prediction performance of only large SVs (> 80 kbp for inversions, > 40 kbp for duplications, > 100 kbp for deletions, and > 100 kbp for translocations). Note that VALOR_1_, LUMPY, DELLY, Sniffles, and Long Ranger are not able to call interspersed duplications, and TARDIS can call duplications < 10 kb, which is smaller than the variants shown in this table. Precision is calculated as $\frac {\text {TP}}{\text {TP+FP}}$, and recall is defined as $\frac {\text {TP}}{\text {TP+FN}}$, where TP is the true positive, FP is the false positive, FN is the false negative, Pr. is the precision, and Rec is the recall. F1-score (shown as F1) is calculated as $2\times \frac {\text {precision}\times \text {recall}}{\text {precision + recall}}$. ^*‡*^SV calls predicted by both Long Ranger and VALOR2 (> 50% reciprocal overlap) are merged into a single call. Best values are highlighted with boldface font

Of the WGS-based tools, Sniffles achieved the highest sensitivity for inversions owing to its use of long reads as it was able to correctly predict 80% of large inversions; however, it suffered from very low precision (11%). On the contrary, using only short reads, TARDIS achieved high precision (97%), but it was able to discover only 38% of the simulated inversions. This is likely because none of the WGS-based tools was optimized to find such large inversion events. VALOR2 showed a very good precision/recall balance with an F1 score of 0.70, but overall, combination of Long Ranger and VALOR2 performed the best in terms of precision/recall for inversions in the simulation experiment.

For large deletions, once again, Long Ranger and VALOR2 combination performed the best, but VALOR2 by itself was able to correctly predict 87% of the simulated variants with a high precision rate (91%). As expected, WGS-based tools (based on both short and long reads) achieved low precision (15 to 46%), although they performed well in terms of recall (78 to 85%).

Finally, the translocation simulation experiment proved VALOR2 to be the best single algorithm in terms of precision with no false-positive calls, with a good recall rate (71%). Only DELLY surpassed VALOR2 in recall (79%), but it suffered from a high number of false positives (26% precision). As in the other experiments, using both Long Ranger and VALOR2 achieved the best F1 score of 95%.

#### Size detection spectrum for structural variation

**Fig. 4:**
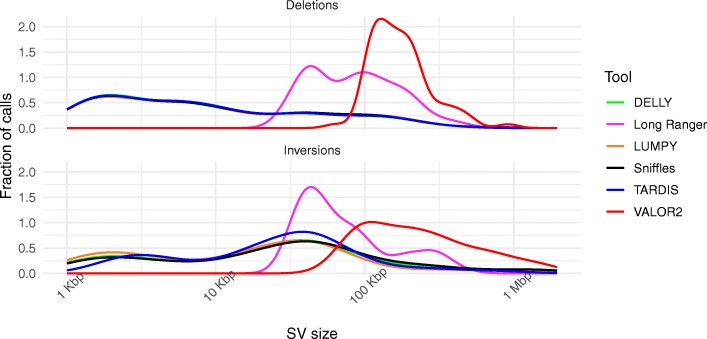
Comparison of size distribution of detected *true* (i.e., known) calls in simulation data as a density plot. We demonstrate that VALOR2 SV detection size range is complementary to WGS-based approaches

As we have described above, our simulation included SVs with different sizes, starting from 50 bp to 6 Mbp. To understand the detection power of using different sequencing technologies, we investigated the size distribution of the correctly identified deletions and inversions in the simulation (Fig. 4). We observe that the both short read-based (TARDIS, DELLY, LUMPY) and long read-based (Sniffles) tools tend to capture similarly sized and relatively shorter SVs compared to the linked-read based (Long Ranger, VALOR2) algorithms. Among the linked-read-based tools, VALOR2 captures larger SVs than Long Ranger, demonstrating its complementary use to Long Ranger, and short- and long-read WGS analysis.

### Biological data sets

Next, we evaluated VALOR2 and compared it to a linked-read-based method (Long Ranger) and three WGS-based tools (DELLY, LUMPY, and TARDIS) using biological data sets. We obtained both linked-read and WGS data from the genomes of a parent-child trio from Yoruba population (NA19238, NA19239, NA19240) [50], one individual of Northern European descent (NA12878) [51], and two haploid genomes (CHM1 and CHM13). The details of the data sources are given in the “Availability of data and materials” section, and we provide large deletion, inversion, and translocation calls generated by VALOR2 in Additional file 1: Tables S2, S3, and S4, respectively. We used DELLY, LUMPY, and TARDIS to generate SV call sets using the WGS data and VALOR2 using the linked-read data on the human reference genome GRCh38. For the haploid genomes, we used VALOR2 in haploid-aware mode, where the read depth and split molecule support thresholds are adjusted accordingly. We obtained the publicly available Long Ranger calls: Yoruba trio call set is available from the Human Genome Structural Variation Consortium [50], and NA12878 call set is available in the European Nucleotide Archive (accession number PRJEB28297), published by Marks et al. [29]. We have run Long Ranger on the CHM1 and CHM13 genomes.

Table 2 summarizes the prediction results of large deletions, segmental duplications (SDs), translocations, and inversions. We note that TARDIS predicts only smaller SDs (< 10 kb), and Long Ranger, DELLY, and LUMPY do not differentiate between tandem and interspersed SDs. We therefore merged different types of SD predictions generated by VALOR2. We also compared our predictions with two different gold standard data sets. For deletions and duplications, we used the non-redundant data set in dbVar [52], and for inversions and translocations, we used gnomAD SV calls [53]. Since gnomAD call set was only available in GRCh37, we used the UCSC liftOver tool to convert the coordinates to GRCh38.

**Table 2:** Large structural variants found in biological data sets

**Variant**	**Sample**	**VALOR2**		**Long Ranger**		**LUMPY**		**DELLY**		**TARDIS**	
		*Pred.*	*Known*^∗^	*Pred.*	*Known*^∗^	*Pred.*	*Known*^∗^	*Pred.*	*Known *^∗^	*Pred.*	*Known*^∗^
Deletions	NA19238	8	8	1	1	81	49	192	127	14	13
	NA19239	10	10	3	3	104	64	232	157	17	14
	NA19240	11	11	2	2	95	59	228	157	15	14
	NA12878	14	14	18	18	138	62	273	170	20	20
	CHM1	9	8	109	72	106	47	226	113	20	19
	CHM13	7	7	95	65	78	43	660	423	10	8
Inversions	NA19238	56	17	2	2	3	0	407	37	14	1
	NA19239	49	15	1	1	4	0	406	33	11	0
	NA19240	89	25	3	2	4	0	435	31	9	1
	NA12878	33	12	5	1	3	0	415	37	43	1
	CHM1	35	26	2	2	3	0	259	23	22	1
	CHM13	40	28	2	2	5	0	1496	65	50	0
Duplications ^*‡*^	NA19238	9	5	3	3	142	91	307	183	77	46
	NA19239	9	5	0	0	158	96	298	189	79	42
	NA19240	19	8	2	2	139	91	284	187	82	47
	NA12878	6	4	20	19	196	93	341	184	293	133
	CHM1	5	3	0	0	164	83	289	138	131	64
	CHM13	7	3	0	0	519	276	1425	784	329	196
Translocations	NA19238	1	0	0	0	336	0	8788	0	N/A	N/A
	NA19239	3	0	0	0	368	0	8946	0	N/A	N/A
	NA19240	1	0	0	0	362	0	9250	0	N/A	N/A
	NA12878	1	0	1	0	842	0	9770	0	N/A	N/A
	CHM1	0	0	0	0	320	0	6511	0	N/A	N/A
	CHM13	0	0	0	0	184	0	117667	0	N/A	N/A

Similar to Table 1, we only report large SVs we discovered in real data sets (> 80 kbp for inversions, > 40 kbp for duplications, > 100 kbp for deletions, and > 100 kbp for translocations). We ran LUMPY using the smoove wrapper as recommended by the authors. Note that TARDIS does not predict translocations. ^*‡*^We merged tandem and interspersed duplications in this table since Long Ranger, LUMPY, and DELLY do not differentiate between them. ^∗^For CNVs (deletions and duplications), known variants refer to those that are reported in dbVar [52] non-redundant call set (https://ftp.ncbi.nlm.nih.gov/pub/dbVar/sandbox/sv_datasets/nonredundant/). For balanced rearrangements (inversions and translocations), we used the gnomAD [53] v2.1.1 call set, lifted over to GRCh38 (https://storage.googleapis.com/gnomad-public/papers/2019-sv/gnomad_v2.1_sv.sites.vcf.gz)

Note that in the absence of complete and curated large SVs that are experimentally validated for these biological data sets, we cannot calculate precision and recall rates. However, assuming the dbVar and gnomAD resources are gold standard, deletion predictions of VALOR2 include no false positives (Table 2). Long Ranger and TARDIS also show low number of false positives for deletions. For inversions, we found that 28 to 70% of VALOR2 calls intersect with previously identified inversions. Although Long Ranger calls intersected better with the gnomAD calls, it also predicted only a handful of inversions. As expected, WGS-based tools showed a higher ratio of likely false positives.

VALOR2 predicts only interspersed segmental duplications (SDs), where Long Ranger, LUMPY, and DELLY can detect only tandem SDs, and TARDIS can detect both, although new location of interspersed SDs should be < 50 kb away from the source. The SDs reported in dbVar are detected using read depth-based methods; therefore, there is no discrimination between interspersed and tandem. Therefore, dbVar only includes the coordinates of the “source copy” of the duplicated segments. We thus compared the source coordinates of our interspersed SD calls with dbVar and found that 43 to 67% of SDs predicted by VALOR2 were previously reported. Only Long Ranger achieved a higher intersection with known data, however with fewer predictions.

Finally, none of the translocation calls predicted by either tool intersects with the gnomAD call set. This is in fact on par with the literature, since no translocations are expected to occur in the germline genomes of healthy individuals as they often play roles in cancer development [54]. Therefore, any translocation predictions are either false positives or could be caused by cell line artifacts [55].

### Functional consequence of predicted variants

A majority of predicted translocations and duplications span regions that do not contain gene coding sequences. This is unsurprising since a large amount of disruptive variants are not expected to be in normal genomes. However, VALOR2 did identify events that potentially affect protein coding genes. A large segmental duplication event at chr1:16,728,420–16,797,669 is present in 5 of the 6 genomes analyzed and found to overlap the *CROCC* gene which encodes a structural component of ciliary motility [56]. Another duplication event covering *CLEC18B* was also found in 3 of 6 genomes. The human C-type lectin 18 is expressed abundantly in various cell contexts in the body [57]. VALOR2 calls also revealed deletion polymorphisms, some of which have been previously characterized, in the human genome (Additional file 1: Table S2). Deletion of *UGT2B17* and *UGT2B28*, genes involved in the metabolism of sex steroid hormones, as well as *OR4F5* (olfactory receptor) were found in at least 3 genomes. These have been previously described as null mutations within the genome [58]. Similarly, only 3 inversion calls overlap protein coding regions in these genomes (Additional file 1: Table S3) though further validation is necessary to confirm functional effect of these SVs on these genes.

## Discussion

Linked-read sequencing techniques emerged very recently and are still developing. Many groups are already realizing the power of these techniques for SV detection and phasing. For example, the InPSYght Consortium has sequenced a schizophrenia case/control cohort of 545 individuals using the 10x Genomics Chromium linked-read technology with the aim to study complex structural variants in a large cohort [59].

While we used the 10xG linked-read datasets to demonstrate the utility of our SV discovery methods, several other linked-read platforms are available. BGI has recently developed a single-tube long fragment read (stLFR) technology (https://www.bgi.com/global/sequencing-services/dna-sequencing/lfr-whole-genome-sequencing/, essentially a linked-read method. The stLFR linked-read technique produces reads longer than 10 kb [60] and BGI plans to make the technique their standard of sequencing in the near future. Several other linked-read platforms are becoming commercially available. In particular, TELL-Seq by Universal Sequencing Technologies (https://www.universalsequencing.com/ is also a recent single-tube linked-read method. TELL-Seq does not require a 10xG-like Chromium instrument and offers a simpler and cheaper library prep routine. Loop Genomics (https://www.loopgenomics.com/) is another developing linked-read method.

PacBio with the release of their Sequel II method and Oxford Nanopore with their newest PromethION have reduced the cost of long-read methods. While it is not prohibitively expensive anymore to generate long reads, the error rate is still much higher compared to short reads and linked-reads. Moreover, long-read protocols cannot be utilized with very low input DNA (e.g., less than 10 ng), which makes ultra-low input linked-read method a very attractive alternative.

In this work, we presented novel algorithms to effectively utilize the encoded long-range information in linked-read data for the purpose of characterizing largescale structural variations. The current state of the art SV detection techniques using linked-read such as Long Ranger or GROC-SVs is optimized for certain range of SV sizes. For example, GROC-SVs achieves the best sensitivity for events in the range of (30–100 kb). However, our technique, VALOR2, can detect events of a size larger than 100 kb, including segmental duplications and translocations. We also demonstrated that VALOR2 is a complementary approach to Long Ranger, and both short and long read-based WGS-based tools for deletion and inversion discovery (Fig. 4). Through simulations, we also showed that VALOR2 is a powerful tool for discovering interspersed segmental duplications and translocations, two of the most difficult and neglected forms of structural variation [13].

A future direction for our study is to integrate additional techniques such as local assembly to characterize smaller-scale SVs (i.e., starting from only 50 bp) and to resolve SV breakpoints more precisely by integrating split reads and local assembly. Local assembly was recently used for detection and assembly of novel sequence insertions using linked-reads [61]. Single-molecule sequencing techniques such as PacBio and Oxford Nanopore (ONT) and long-range genome mapping techniques at single-molecule resolution such as Bionano Genomics are becoming more developed and cost effective. We can explore single-molecule techniques not only for the purpose of further validation of our algorithms but also for devising integrative computational techniques to fully resolve the complexity of repetitive DNA common in mammalian genomes.

## Supplementary information

**Additional file 1** Algorithm S1, Figure S1-S2, Table S1-S5.

**Additional file 2** Review history.

## Footnotes

1 For example, 30 × sequence coverage corresponds to 150 × physical coverage when molecule coverage is only 0.2 ×.

2 Note that the empirical value of *μ*_molecule_ is calculated after the molecule recovery step. Therefore, here, we use *μ*_molecule_ as the *expected* value and set to 40,000 by default (can be changed by the user).
