# Supplementary material for: VALOR2: characterization of large-scale structural variants using linked-reads
Source: Genome Biol. 2020 Mar 19;21:72. doi: 10.1186/s13059-020-01975-8 (PMC7083023; doi:10.1186/s13059-020-01975-8)
Supplement: Supplementary file 1 — Additional file 1 Algorithm S1, Figure S1-S2, Table S1-S5. [file 13059_2020_1975_MOESM1_ESM.pdf]

Supplementary Information

Submolecule detection algorithm

Algorithm S1 Submolecule detection.

Require: Alignments in BAM format with barcodes, look-ahead parameter (Q), extend parameter (T).

Ensure: Set of submolecules $S_M = \{M_1, M_2 \ldots, M_k\}$ (value of k is unknown and will be determined by the algorithm)

```
S_M ← ∅
i ← 1
for each chromosome c do
    for each barcode b do
        M_i = ∅
        for l = 1 to length(c) do
            if short fragment f with barcode b maps to c[l] then
                if M_i = ∅ then
                    M_i ← f
                    s(M_i) ← s(f)
                    e(M_i) ← e(f)
                else if (s(f) < s(M_i) + Q) or (s(f) < e(M_i) + T) then
                    M_i ← M_i ∪ f
                    e(M_i) ← e(f)
                else
                    S_M ← S_M ∪ M_i
                    i ← i + 1
                end if
            end if
        end for
    end for
end for
return S_M
```

$s(f)$ denotes the map start location and $e(f)$ denotes the map end location of fragment f.

Supplementary Figures

a)

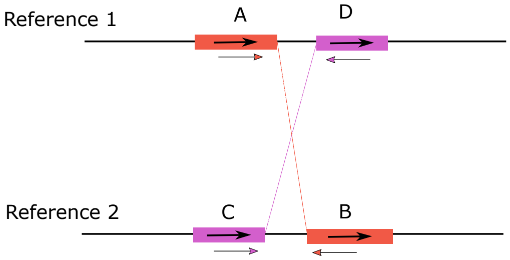

b)

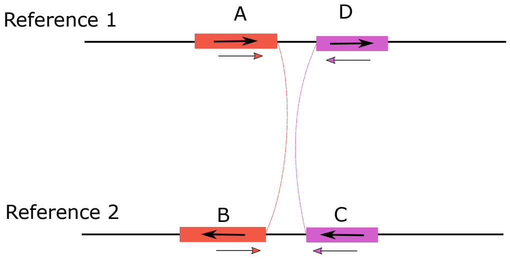

Figure S1: Split molecule and read pair sequence signatures used to discover a) Direct and b) Inverted reciprocal translocation breakpoints in VALOR2.

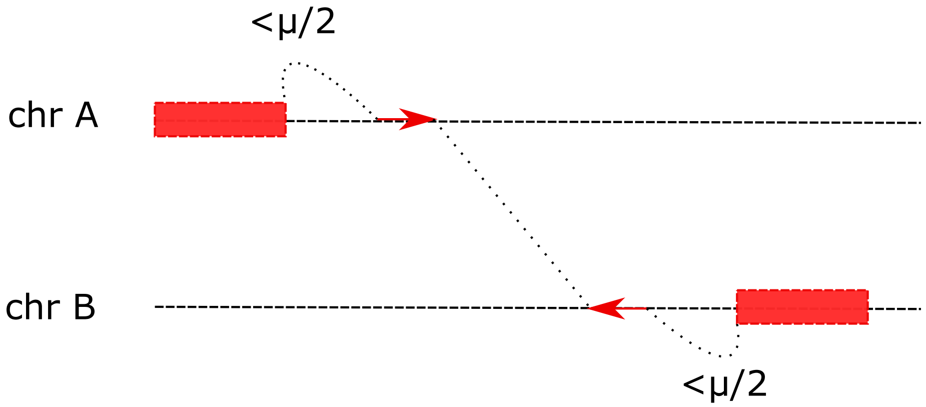

Figure S2: Forming candidate splits for inter-chromosomal variants.

Table S1: Versions and parameters used for the tools in benchmarks.

Tool	Version	Sample Command Line
VALOR2	2.1.5	valor2 -i input.bam -s GRCh38.sonic -o output (female samples)
VALOR2	2.1.5	valor2 -i input.bam -s GRCh38.sonic -o output -y X -y Y (male samples)
VALOR2	2.1.5	valor2 -i input.bam -s GRCh38.sonic -o output -p 1 (haploid genomes)
LongRanger	2.2.0	longranger –vcmode=freebayes –project v5LongrangerSV –reference refdata-human_g1k_v37/ –fastqs=. –id=10Xv5SV –sex=MALE
LongRanger	2.2.0	longranger –vcmode=freebayes –project v5LongrangerSV –reference refdata-human_g1k_v37/ –fastqs=. –id=10Xv5SV –sex=FEMALE
VALOR	1.0.0	READ_LENGTH: 150, FRAG_SIZE: 1,000, CLONE_MEAN: 50,000, CLONE_STD_DEV: 10,000, INV_MIN_SIZE: 40,000, INV_MAX_SIZE: 10,000,000, INV_GAP: 1,000, INV_OVERLAP: -1,000, QCLIQUE_LAMBDA: 0.5, QCLIQUE_GAMMA: 0.6
DELLY	0.8.1	delly call -g ref.fasta -o output.bcf input.bam
LUMPY/smoove	0.2.13/0.2.4	smoove call –outdir dir –exclude exclude.cnvnator_100bp.GRCh38.20170403.bed –name sample_name –fasta ref.fasta -p num_threads –genotype input.bam
TARDIS	1.0.7	tardis -i input.bam –ref ref.fasta –sonic GRCh38.sonic –first-chr 0 –last-chr 24 –out tardis
SNIFFLES	1.0.7	sniffles -m input.bam -v output.vcf

The GRCh38 SONIC file required by VALOR2 and TARDIS is available at `https://github.com/BilkentCompGen/sonic-prebuilt`. The exclusion coordinates required by LUMPY/smoove is available at `https://github.com/hall-lab/speedseq/blob/master/annotations/exclude.cnvnator_100bp.GRCh38.20170403.bed`.

Table S2: Large deletions found in biological data sets using VALOR2.

**Coordinates**				**Predictions**					
chrom.	start	end	# genes	CHM1	CHM13	NA12878	NA19238	NA19239	NA19240
1	29764	181077	1	yes	yes	yes			
1	120362054	120915183	2				yes		
1	121616887	121827452	0	yes			yes		
1	143307225	143502174	0			yes	yes		
1	196762025	196842651	2					yes/valid	
2	88860579	89142989	0			yes			yes
2	88861538	89245252	0			yes			
2	241909597	242095162	0	yes					
3	162793600	162909522	0	yes		yes	yes	yes/valid	
3	191504818	191619525	0		yes				
4	68507824	68625997	1			yes		yes/valid	yes
4	69258996	69368703	1	yes	yes	yes			
5	69623516	70449210	2						yes
5	69710160	69849637	0						yes
5	69992199	70147000	2						yes
7	76452379	76582479	3				yes		
8	7772400	8017996	11			yes			
8	39373595	39530805	0	yes		yes			
9	42586198	42727642	0	yes		yes			
11	55652747	55838034	8					yes/valid	
12	9478492	9582868	0		yes			yes/valid	yes/valid
15	24215185	24315498	0				yes/valid		
15	34419109	34537019	2				yes	yes	
16	29445427	29530727	5				yes		
16	32123144	32799318	2					yes	yes
17	3107335	3218882	3						yes
17	38101679	38217194	2				yes		
17	38125655	38200401	2		yes				
17	46307479	46490609	2	yes		yes		yes	
19	20412145	20535865	1			yes			
20	25775788	25851053	0					yes/valid	yes/valid
22	18773479	18870560	0				yes		yes
22	21195022	21313496	1					yes	
X	4570154	4890440	0	yes					

Table S3: Large inversions found in biological data sets using VALOR2.

Coordinates				Predictions					
chrom.	start	end	# genes	CHM1	CHM13	NA12878	NA19238	NA19239	NA19240
1	16728420	16797669	1	yes	yes	yes	yes		yes
7	62330741	62444256	0			yes		yes	yes
9	39391755	39537339	0			yes			
9	63858198	63917112	0					yes	yes
16	21517246	21582834	0						yes
16	34586125	34879925	0		yes			yes	yes
16	74354098	74426665	2	yes	yes				yes
18	15472414	15564649	0						yes
20	131052026	31105662	0	yes	yes				
20	31168545	31230728	0		yes			yes	yes
20	31179710	31242313	0			yes		yes	yes
20	631183681	31269731	1				yes		
21	8386027	8472360	0						yes
22	12501442	12601105	0				yes	yes	
Y	11285469	11548942	0					yes	
Y	11293286	11325082	0	yes					
Y	11292302	11313722	0		yes				
Y	10755775	10800602	0	yes					
Y	20072261	20189708	0					yes	
Y	20261006	20358747	0					yes	

Table S4: Translocations found in biological data sets using VALOR2.

**Cooordinates**								**Predictions**					
source			destination										
chr	start	end	chr	start	end	Is reciprocal	# genes	CHM1	CHM13	NA12878	NA19238	NA19239	NA19240
2	94517792	94567521	21	12974065	13060337	yes	0			yes			
2	94523229	94567885	9	43153430	43215547	yes	0			yes			
4	49106801	49149624	5	49657239	49661147	yes	0				yes	yes	yes
4	49099395	49149773	5	49657318	49660040	yes	0				yes	yes	
4	49096330	49154583	17	21972211	21975277	yes	0				yes	yes	
4	49151388	49249730	2	89827546	89835165	no	0			yes			
4	49151388	49249762	2	89828952	89831103	no	0			yes			
4	49152168	49251084	17	21971403	21974443	no	0			yes			
4	49151539	49246651	20	31185500	31186311	no	0			yes			
4	49134928	49153538	20	31185693	31196126	yes	0					yes	
7	158567911	158618939	22	43495203	43500244	yes	0	yes					
9	376270	426497	22	43495391	43500046	yes	1	yes					
9	65051883	65075271	18	15547917	15659112	yes	0					yes	
10	41877082	41895332	2	89828098	89834976	yes	0			yes			
10	41874455	41899331	4	49644164	49646447	yes	0			yes			
10	41872311	41898914	4	49648378	49649535	yes	0						yes
10	41875047	41907941	5	49657322	49660485	yes	0					yes	
10	41869925	41908141	20	31106720	31106770	yes	0				yes		
10	41868606	41911425	17	21969829	21972090	yes	0			yes			
10	41876979	41896561	2	89829808	89836094	yes	0			yes			
11	147165	235452	17	113542	144403	yes	1	yes	yes		yes		yes
14	18236622	18304314	13	16799090	16861807	yes	0					yes	
16	12036	81765	X	156008598	156029932	yes	1			yes			
16	34095132	34175643	20	30924654	30953938	no	0						yes
16	34095133	34175643	17	26936837	26940849	no	0						yes
16	34128363	34227613	Y	10918370	10920884	no	0					yes	
16	34128363	34227613	Y	10927484	10928807	no	0					yes	
16	34067355	34093702	Y	10892197	10893938	yes	0					yes	
16	34070138	34095601	Y	10905986	10906555	yes	0					yes	
16	46401797	53273293	1	125178171	125183744	no	32				yes		
17	136863	201227	11	101346	128318	yes	0	yes	yes	yes	yes		yes
17	146898	212103	11	140823	146501	yes	0					yes	
17	101899	220313	11	82499	218805	yes	1					yes	
17	26821599	26830298	21	7923582	7957680	yes	0					yes	
18	15372590	15410606	9	65049525	65063105	yes	0				yes		
18	80206786	80263178	4	190122540	190123056	yes	0				yes		
19	1528857	1569012	11	69254640	69285047	yes	0	yes					
20	30919820	30993610	16	34165769	34191135	yes	0			yes			yes
20	31185722	31200944	4	49135417	49153328	yes	0					yes	
21	7247265	7292331	Y	10919841	10919976	yes	0		yes				
21	12970370	13050652	18	15351150	15407232	yes	0						yes
22	11328338	11372973	13	16250613	16255546	yes	0					yes	yes
22	43496173	43500188	7	158562445	158623893	yes	1	yes					
X	2741437	2826890	Y	2797570	2817054	yes	0					yes	
X	156011548	156026579	16	104377	112373	yes	0					yes	

Table S5: Sources of biological data used in this project and the call sets.

Genome	Sequencing Tech	Sequencing data	Citation	Call set	Link
NA12878	Illumina WGS	ENA Accession PRJEB3381	Illumina Platinum Genomes	TARDIS	doi:10.5281/zenodo.3380054
				DELLY	doi:10.5281/zenodo.3380054
				LUMPY	doi:10.5281/zenodo.3380054
	10x Genomics	ENA Accession PRJEB28297	Marks et al., 2019	VALOR2	doi:10.5281/zenodo.3380054
				Long Ranger	https://www.ebi.ac.uk/ena/ /browser/view/PRJEB28297
YRI trio	Illumina WGS	http://ftp.1000genomes.ebi.ac.uk /vol1/ftp/data_collections/ /hgsv_sv_discovery/data/YRI/	HGSV	TARDIS	doi:10.5281/zenodo.3380054
				DELLY	doi:10.5281/zenodo.3380054
				LUMPY	doi:10.5281/zenodo.3380054
	10x Genomics	http://ftp.1000genomes.ebi.ac.uk/ /vol1/ftp/data_collections/ /hgsv_sv_discovery/working/ /20160513_10XGenomics_data_and_calls/	HGSV	VALOR2	doi:10.5281/zenodo.3380054
				Long Ranger	http://ftp.1000genomes.ebi.ac.uk/ /vol1/ftp/data_collections/ /hgsv_sv_discovery/working/ /20160513_10XGenomics_data_and_calls/
CHM1	Illumina WGS	NCBI Accession SRX652547	Audano et al., 2019	TARDIS	doi:10.5281/zenodo.3380054
				DELLY	doi:10.5281/zenodo.3380054
				LUMPY	doi:10.5281/zenodo.3380054
	10x Genomics	https://support.10xgenomics.com/ /de-novo-assembly/datasets/2.0.0/chm		VALOR2	doi:10.5281/zenodo.3380054
				Long Ranger	doi:10.5281/zenodo.3380054
CHM13	Illumina WGS	NCBI Accession SRX6770568	T2T Consortium	TARDIS	doi:10.5281/zenodo.3380054
				DELLY	doi:10.5281/zenodo.3380054
				LUMPY	doi:10.5281/zenodo.3380054
	10x Genomics	https://github.com/ /nanopore-wgs-consortium/CHM13	T2T Consortium	VALOR2	doi:10.5281/zenodo.3380054
				Long Ranger	doi:10.5281/zenodo.3380054
