# Supplementary material for: VALOR2: characterization of large-scale structural variants using linked-reads
Source: Genome Biol. 2020 Mar 19;21:72. doi: 10.1186/s13059-020-01975-8 (PMC7083023; doi:10.1186/s13059-020-01975-8)
Supplement: Supplementary file 2 — Additional file 2 Review history. [file 13059_2020_1975_MOESM2_ESM.docx]

**First round**

**Reviewer 1**

**Are the methods appropriate to the aims of the study, are they well described, and are necessary controls included?**

Yes

**Are the conclusions adequately supported by the data shown?**
Yes

**Are sufficient details provided to allow replication and comparison with related analyses that may have been performed?**

Yes

**Does the method perform better than existing methods (as demonstrated by direct comparison with available methods)?**

Yes

**Is the method likely to be of broad utility? Is any software component easy to install and use?**

The method is of utility to researchers interested in discovering long duplications and inversions using 10XGenomics data.

**Is the paper of broad interest to others in the field, or of outstanding interest to a broad audience of biologists?**

No

This paper presents a method to discover segmental duplications, inversions, and, in combination, inverted duplications, based on 10X Genomics (10XG) data. 10XG data are generated by barcoding long (in the 10k order of magnitude range) DNA molecules, pooling several of them, finally fragmenting the molecules in one pool, and sequencing the resulting fragments using Illumina. Upon sequencing, the barcodes allow the user to stitch fragments together to obtain a more comprehensive picture of the genome that is analyzed.

To demonstrate the achievements from a larger perspective, the authors present several results where, most importantly, VALOR2 (the new algorithm) outperforms LUMPY and DELLY, which represent state-of-the-art algorithms.

There are the following major issues:

- What about comparing against (read depth based) copy number variant (CNV) discovery tools? Those are able to spot large CNV's. It would be great if one could understand the gain also relative to such methods.

- How does VALOR2 embed with current efforts in terms of sequencing protocols that aim at the particular discovery of duplications and/or inversions, or, at least, whose protocols particularly support their discovery? [I know there are, but the names do not come to my mind]

- On simulated data, Long Ranger seems to be worse in Recall, while on real data, it happens to achieve (overproportionally) better Recall? Why is this? This tends to create some mistrust in the data simulation procedure -- please try to explain what is going on.

- The Discussion is not written appropriately. A Discussion should address the hypotheses raised in the Introduction and discuss to what degree the hypotheses have been confirmed by the Results presented.

- The Results are rather little, and focus nearly entirely on outperforming other tools. Anything surprising in terms of what kind of duplications / inversions can be spotted with VALOR2, but not with previous tools? Can the new duplications/inversions found be characterized a bit?

- The Methods part is rather fuzzy in terms of definitions (in particular the definition of a 'submolecule'), and therefore hard to follow at times.

- Are LUMPY and DELLY really the currently best tools available? For what I remember there is recent work improving on these tools with respect to duplications and inversions: please doublecheck papers that cite LUMPY and DELLY, and which claim to raise improvements.

- What do improvements of VALOR2 over former versions ('VALOR1') look like? The reader really wants to know. Not only a theoretical explanation, but also a practical evaluation is required.

- The algorithm presented in the Methods part is (very) straightforward. While simplicity is a good thing in general, the manuscript does not deserve to be published based on the methods presented in their own right. So, Results and Discussion are decisive, which means that one needs to possibly improve substantially on those two parts.

Some minor issues:

- please sort citations by number within square brackets (I was taught that this was standard practice)

- p7 - l25: What's the definition of 'IH'?

- Definition of 'submolecule' rather unclear

- p10 - l10: the underlying large molecules are called submolecules? If so, the word 'submolecule' is a misleading. Sentence seems to be broken (and unfortunately introduces some confusion)

- p10 - l16: definition of span? Hard to follow what the pairing means, illustrations or better definitions necessary

- p10 - l19: what is a "candidate split with no read pair support"?

- Figure 3: Explanations are missing, hard to follow. (and in (c) shouldn't map breakpoints the other way round? -- if not, what's the difference with (b)?)

**Reviewer 2**

**Are the methods appropriate to the aims of the study, are they well described, and are necessary controls included?**

Yes

**Are the conclusions adequately supported by the data shown?**
Yes

**Are sufficient details provided to allow replication and comparison with related analyses that may have been performed?**

Yes

**Does the method perform better than existing methods (as demonstrated by direct comparison with available methods)?**

Yes

**Is the method likely to be of broad utility? Is any software component easy to install and use?**

The tool is easy to download and install.

I suggest comparing the VALOR2 method developed for linked-reads to a method recently developed for Illumina short reads by the same team (TARDIS). TARDIS detects similar types of stuctural variants as VALOR2 using a different approach and based on different types of evidence for structural variants.

**Is the paper of broad interest to others in the field, or of outstanding interest to a broad audience of biologists?**

No

The paper presents an algorithm, VALOR2, to detect segmental duplications and large inversions from so-called linked-reads (i.e., Illumina short reads data with additional long-range information). The algorithm is an improvement of the VALOR methods published in BMC Genomics in 2017. The algorithm has been extensively validated on several different data sets. And the statistics in the manuscript are used correctly.

Major comment:

1. The VALOR2 method solve an important problem of identifying three types of structural variants: tandem and interspersed duplications and inversions. Given the expertise of the authors in the domain of SV detection, I would expect their method to be able to identify other typical SVs such as deletions, translocations, etc. Can the authors integrate detection of these SVs into their method?

Minor comments:

1. In the introduction the authors state that "no method yet exists to anchor a new SD (i.e. ﬁnd their insertion locations)", which is not actually true, at least for simple Illumina paired-end reads (e.g., TARDIS from the same team). I suggest that the authors reformulate the sentence by saying that "no method using linked-reads yet exists.."

2. It seems that the recall value for VALOR2 for inversions is not correctly calculated. According to the provided numbers it should be 0.68 instead of 0.76.

3. The authors compare their method with DELLY and LUMPY developed for Illumina small read data. Should not they compare VALOR2 with their own TARDIS method for the discovery of complex SVs?

4. Can the authors make available the simulated data in addition to other datasets they used for the algorithm validation?

**Response to reviewers**

**Reviewer 1**

What about comparing against (read depth based) copy number variant (CNV) discovery tools? Those are able to spot large CNV's. It would be great if one could understand the gain also relative to such methods.

Read depth based methods can find CNVs, however, they cannot predict the new insertion location, or the inverted/direct status. We aimed to highlight this fact in the introduction: “Note that it is still possible to identify increase in SD copy number using read depth signature [3, 40], however, no method yet exists to anchor a new SD (i.e. find their insertion locations).”

How does VALOR2 embed with current efforts in terms of sequencing protocols that aim at the particular discovery of duplications and/or inversions, or, at least, whose protocols particularly support their discovery? [I know there are, but the names do not come to my mind]

We are not aware of other sequencing protocols that particularly support duplication discovery, and to our knowledge only short, long, and linked-read sequencing data sets exist for the genomes that we analyzed. We are aware of StrandSeq protocol for inversion discovery (Sanders et al, Genome Res 2016), however, no data or call sets are available for the genomes we have tested. We also compare our calls to dbVar (which also includes HGSV) and gnomAD call sets, which integrate SV calls using various sequencing platforms and algorithms.

On simulated data, Long Ranger seems to be worse in Recall, while on real data, it happens to achieve (overproportionally) better Recall? Why is this? This tends to create some mistrust in the data simulation procedure -- please try to explain what is going on.

We were not able to calculate recall for real data since there are no established gold-standard large SV call sets for NA12878 genome so that type of result was not presented in the manuscript, therefore we are confused about the reviewer’s comment. Reviewer 1 also asks for more explanation about the simulation, which we have now added to the manuscript. Briefly, we used the standard SV simulation tool, VarSim. We also point out that VarSim “inserts” SVs that are previously reported in various databases such as dbVar and DGV, therefore all simulated SVs are variants that are previously discovered and validated in many other genomes . More details can be found in the manuscript now.

The Discussion is not written appropriately. A Discussion should address the hypotheses raised in the Introduction and discuss to what degree the hypotheses have been confirmed by the Results presented.

We have rewritten the Discussion section entirely.

The Results are rather little, and focus nearly entirely on outperforming other tools. Anything surprising in terms of what kind of duplications / inversions can be spotted with VALOR2, but not with previous tools? Can the new duplications/inversions found be characterized a bit?

Since we have new experiments and repeated the older simulations, all our results have changed. We also added a new subsection (3.3. Functional consequence of predicted variants) to address novel biological characterizations.

The Methods part is rather fuzzy in terms of definitions (in particular the definition of a 'submolecule'), and therefore hard to follow at times.

We would like to note that we have a Glossary subsection in the Methods section that also includes the definition of submolecule among other terminology we used in the manuscript. We have now improved this section, and provided examples in Figure 1.

Are LUMPY and DELLY really the currently best tools available? For what I remember there is recent work improving on these tools with respect to duplications and inversions: please double check papers that cite LUMPY and DELLY, and which claim to raise improvements.

They are the most commonly used tools and are part of the 1000 Genomes Human Genome Structural Variation Consortium (Chaisson et al, Nat Comm, 2019). We also now added our own TARDIS tool as proposed by Reviewer 2, and Sniffles, which is an SV caller that uses long reads.

What do improvements of VALOR2 over former versions ('VALOR1') look like? The reader really wants to know. Not only a theoretical explanation, but also a practical evaluation is required.

VALOR1 predicts only inversions using pooled clone sequencing data (Kitzman et al 2011). On the other hand VALOR2 is developed solely for 10x Genomics Linked-Reads and it also predicts duplications, deletions and translocations. We have now added VALOR1 results to the simulation experiment, and clearly explain the improvements of VALOR2 over VALOR1 in the Introduction. We copy the text below:

“Briefly, VALOR 2 differs from the former version of VALOR through: 1) it can characterize segmental duplications in both direct and inverted orientation, 2) it can discover translocations and deletions, 3) it incorporates read depth information to improve predictions and reduce false calls, 4) it provides full support to alignment files (i.e., BAM) generated from 10xG Linked-Read data sets, and 5) provides a 10-fold speed up in run time (data not shown).”

The algorithm presented in the Methods part is (very) straightforward. While simplicity is a good thing in general, the manuscript does not deserve to be published based on the methods presented in their own right. So, Results and Discussion are decisive, which means that one needs to possibly improve substantially on those two parts.

We agree with the reviewer that simplicity is a good thing. Formulations used in VALOR2 still give rise to an NP-hard problem (maximal clique), therefore the details are not very simple. Currently the large SV discovery still is not studied well, and especially the use of Linked-Reads are still very new. Therefore we believe that our methods still deserve to be published on the basis that they try to address an unsolved problem, provide good accuracy, and aim to fill an important gap in our ability to characterize large genomic variation.

**Reviewer 2**

The paper presents an algorithm, VALOR2, to detect segmental duplications and large inversions from so-called linked-reads (i.e., Illumina short reads data with additional long-range information). The algorithm is an improvement of the VALOR methods published in BMC Genomics in 2017. The algorithm has been extensively validated on several different data sets. And the statistics in the manuscript are used correctly.

We thank the reviewer for their comments.

Major comment:

1. The VALOR2 method solve an important problem of identifying three types of structural variants: tandem and interspersed duplications and inversions. Given the expertise of the authors in the domain of SV detection, I would expect their method to be able to identify other typical SVs such as deletions, translocations, etc. Can the authors integrate detection of these SVs into their method?

We thank the reviewer for acknowledging our expertise in SV detection. We have now integrated formulations and methods to detect large deletions and translocations (both reciprocal and arm-switch) in the new version of VALOR2, and demonstrate its use using new simulations and real whole genome data sets.

Minor comments:

1. In the introduction the authors state that "no method yet exists to anchor a new SD (i.e. nd their insertion locations)", which is not actually true, at least for simple Illumina paired-end reads (e.g., TARDIS from the same team). I suggest that the authors reformulate the sentence by saying that "no method using linked-reads yet exists.."

We thank the reviewer for the comment. We fixed the relevant sentence.

2. It seems that the recall value for VALOR2 for inversions is not correctly calculated. According to the provided numbers it should be 0.68 instead of 0.76.

We thank the reviewer for finding this mistake. We corrected all precision and recall values.

3. The authors compare their method with DELLY and LUMPY developed for Illumina small read data. Should not they compare VALOR2 with their own TARDIS method for the discovery of complex SVs?

We omitted TARDIS in the first version as the size range of SVs detected by VALOR2 and TARDIS are complementary. TARDIS aims to discover smaller (< 50 Kb) structural variation, and VALOR2 aims to find larger (> 50 Kb). However, in this version we now added TARDIS in our benchmarks.

4. Can the authors make available the simulated data in addition to other datasets they used for the algorithm validation?

We uploaded all VCF and BAM files to Zenodo (doi:10.5281/zenodo.3380054

**Second round**

**Reviewer 2**

All methods are appropriate to the aims of the study, and they are well described. The authors implement a novel and interesting idea of quasi-clique discovery in graphs describing putative structural variants. Moreover, to identify duplications, the authors propose to complement the read signatures with read coverage information.

The paper has been greatly improved since the last submission. The authors have indeed addressed all reviewers' comments. In particular, the authors have added to the program the possibility to detect most types of large structural rearrangements, which made of their method a very useful tool for the community. Importantly, for the ease of the installation, the authors provide a docker container.

Conclusions of the paper are adequately supported by the data shown. The authors compare their method with the most popular and/or novel structural variant discovery tools, such as LUMPY, Delly, Long Ranger, TARDIS and Sniffles.

Minor comments:

Could the authors provide information about the parameters used to test each method to allow replication of results?

p.4. Par. 4. Why didn't the authors provide statistics about deletions?

Figure 4. The quality of the figure can be improved (line thickness, similar colors for Valor and Delly, disproportionally large Y-axis).

Results 3.3. This part of Results (functional consequences) is written in a very sketchy way (esp. see line 4). Can the authors extend and improve this subsection?

**Response to reviewer**

Could the authors provide information about the parameters used to test each method to allow replication of results?

We now included a new supplementary table (Additional file 1: Table S1) that includes tool version tables and the parameter sets used for each tool.

p.4. Par. 4. Why didn't the authors provide statistics about deletions?

We thank the reviewer for pointing out this oversight. We have now added the deletion statistics to the relevant sentence.

Figure 4. The quality of the figure can be improved (line thickness, similar colors for Valor and Delly, disproportionally large Y-axis).

We have now edited the Figure 4 by increasing line thickness, changing colors, and rescaling the Y-axis.

Results 3.3. This part of Results (functional consequences) is written in a very sketchy way (esp. see line 4). Can the authors extend and improve this subsection?

Since the biological samples we tested in this manuscript carry no known genetic disease, we could not find many functionally important and/or deleterious variation. However, we still extended and improved this section.
